# LC–MS/MS quantitative analysis of phylloquinone, menaquinone-4 and menaquinone-7 in the human serum of a healthy population

**DOI:** 10.7717/peerj.7695

**Published:** 2019-09-19

**Authors:** Katerina Dunovska, Eva Klapkova, Bruno Sopko, Jana Cepova, Richard Prusa

**Affiliations:** Department of Medical Chemistry and Clinical Biochemistry, Charles University Prague, Second Faculty of Medicine and University Hospital Motol, Prague, Czech Republic

**Keywords:** Phylloquinone, Menaquinone, Deuterated internal standard, LC–MS/MS

## Abstract

A novel application of the liquid chromatography method combined with the triple quadrupole tandem mass spectrometry method was developed for the quantification of vitamin K_1_ and two forms of vitamin K_2_ (menaquinone-4, menaquinone-7) in human serum. Total chromatography time for each run was 9 min. Time required for the sample pretreatment procedures was approximately 4 h. The coefficients of variation (CVs) of intra-assay were 10.4%, 3.2 % and 2.3% for vitamin K_1_ in three levels of quality control samples; were 14.3%, 3.2% and 6.7% for menaquinone-4; and were 11.1%, 6.0% and 7.0% for menaquinone-7. The inter-assay CVs were 12.8%, 11.3% and 7.4% for vitamin K_1_; were 15.2%, 9.2% and 8.7% for menaquinone-4; and were 13.2%,11.1% and 7.2% for menaquinone-7. No interference was found between K_1_, menaquinone-4 and menaquinone-7, nor any deuterated internal standards. This method was then used to determine reference values for Caucasian populations of central European origin. Samples were measured from 191 healthy volunteers (51.2 ± 16.2 years (mean ± SD)) and the values concerning K_1_ were 0.044–1.357 ng/mL for women and 0.030–1.214 ng/mL for men. The values for menaquinone-4 and menaquinone-7 did not exhibit any differences between women and men, and were 0.050–1.598 and 0.074–0.759 ng/mL, respectively.

## Introduction

Vitamin K is a cofactor for enzymatic modification of glutamic acid residues (Glu) to gamma-carboxyglutamic acid residues (Gla) in vitamin K-dependent Gla proteins. These Gla proteins are necessary for hemostasis, bone metabolism, vascular calcification and cell proliferation. Vitamin K is found in nature as phylloquinone (vitamin K_1_) in green leafy vegetables, algae and some plant oils, and menaquinones (vitamin K_2_ also termed MK-*n*, where *n* is the number of isoprenoid units) can be found in meat, eggs and fermented food. A synthetic form of vitamin K menadione (vitamin K_3_) can be found as an additive in some animal feeds. Recently, new synthetic vitamin K forms have been described: vitamin K_4_ (menadiol sodium phosphate), which is the water-soluble form derived from menadione by reduction; and vitamin K_5_ (4-amino-2-methyl-1-naphtol). Vitamin K_1_ is the main form of vitamin K in the Western diet (about 90%), whereas menaquinones make up about 10%. This form of vitamin K is important mainly for blood coagulation. The main functions of vitamin K_2_ were discovered to be bone metabolism, vascular calcification and cell proliferation. In some studies, vitamins K_2_, K_3_, K_4_ and K_5_ have been shown to have anticancer effects (Fu et al., 2009; Jiang et al., 2013; Lee et al., 2015; Nakaoka et al., 2015; Osman, El-Abd & Nasrallah, 2016; EFSA NDA Panel (EFSA Panel on Dietetic Products, Nutrition and Allergies), 2017).

As a cofactor to carboxylase, vitamin K undergoes a cycle of oxidation and reduction that allows for its reuse. The enzyme that modifies the vitamin K-dependent proteins is vitamin K gamma glutamyl carboxylase. The carboxylated Glu residues are turned into Gla amino acids, and a reduced vitamin K molecule is converted to a vitamin K epoxide (KO). Before it can be reused, this vitamin K epoxide has to be converted back to reduced vitamin K (hydroquinone) by vitamin K epoxide reductase (VKOR) in a two-step reaction. At first, vitamin KO is converted to vitamin K by the VKOR. The second step is catalyzed by either VKOR or possibly another yet-to-be-defined reductase. Those two steps are sensitive to vitamin K antagonists such as warfarin. There is one warfarin-resistant pathway where the reduction of vitamin K to hydroquinone is catalyzed by a NAD(P)H-dependent reductase (Stafford, 2005; Tie & Stafford, 2015). Tie et al. (2011, 2013) discovered that in situations when VKOR is inactivated by warfarin or knocked out by gene targeting in HEK293 cells, those cells can still efficiently reduce vitamin K to support vitamin K-dependent carboxylation. The half-lives of vitamin K forms are different. Phylloquinone and menaquinone-4 (MK-4) have the shortest half-life, about 1.5–2.0 h. The half-life of menaquinones with longer side chains is around 72 h (Food and Agriculture Organization of the United Nations/World Health Organization, 2001).

Recommended daily intakes (RDIs) of vitamin K are very variable. In the US, the recommended adequate intake (AI) of vitamin K is 120 µg/day for men and 90 µg/day for women. The Italian Society of Human Nutrition suggested that the AI of vitamin K should be 140 µg/day for people 18–59 years old and 170 µg/day for people >60 years old. In the UK, the AI of vitamin K has not been precisely established. Previous data calculated AI using one µg/kg body weight. This amount might be adequate for blood coagulation, but it is insufficient for bone metabolism and vascular calcification (Fusaro et al., 2017). In the Czech Republic, the RDI for vitamin K is reported as 75 µg.

The most commonly used methods for determination of vitamin K levels are high performance liquid chromatography (HPLC) with post-column reduction and fluorescent or electrochemical detection, but these methods require extensive sample pre-purification due to interference of lipids. The newest methods are based on the principle liquid chromatography method used with tandem mass spectrometry (LC–MS/MS) methods, which should be more sensitive and specific with a lower limit of detection (Fusaro et al., 2017).

## Survey methodology

### Materials and Methods

#### Reagents

Liquid chromatography–mass spectrometry-grade methanol, HPLC-grade ethanol, LC–MS-grade isopropanol, n-hexane, diethyl ether, ammonium fluoride, and analytical standards of: phylloquinone, MK-4 and MK-7, were obtained from Supelco and Honeywell (Labicom, Olomouc, Czech Republic). Deuterium-labelled internal standards of the vitamins (d7-MK-7, d7-MK-4 and d7-K_1_) were obtained from Toronto Research Chemicals Inc. (Toronto, ON, Canada).

#### Equipment

We used an Agilent Technologies 1290 Infinity II LC system, including an autosampler, binary pumps and a thermostatted column compartment with 6470 Triple Quad (Agilent Technologies, Santa Clara, CA, USA). Other necessary equipment were MS 40+ Vacuum Products (Agilent Technologies, Santa Clara, CA, USA) and a nitrogen generator NM32LA (Peak Scientific, Inchinnan, UK). The sample separation was carried out on a reversed phase column SB-C8, 1.8 µm, 2.1 × 100 mm (Agilent Technologies, Santa Clara, CA, USA).

#### Liquid chromatography conditions

The column was operated at 30 °C. A chromatographic separation was achieved under the gradient flow of eluents, initially at 15/85 mix of mobile phase [A] water and methanol (50/50, v/v with 0.1% ammonium fluoride) and [B] methanol (100% with 0.1% ammonium fluoride). The gradient set up is shown in Table 1. The autosampler was cooled at 7 °C.

**Table 1:** Gradient of mobile phases.

Time (min)	A (%)	B (%)	Flow (mL/min)
0	15	85	0.40
1	15	85	0.40
8	0	100	0.40
9	0	100	0.50
9.1	0	100	0.60
11.5	0	100	0.60

#### Mass spectrometry conditions

The Agilent Jet Stream with electrospray ionization (ESI) ion source operated in positive ion mode. The scan type used dynamic multiple reaction monitoring (MRM). The measurements used were: gas temperature at 340 °C, gas flow eight L/min, nebulizer at 28 psi, sheath gas temperature 380 °C, and sheath gas flow 12 L/min. An overview of the MRM transitions, collision energies and retention times for the analytes are given in Table 2.

**Table 2:** Mass spectrometry conditions—transitions, retention times, collision energies (the analytes marked by * were used for quantification).

Compound name	Precursor ion	Product ion	RT (min)	Collision energy
d7-K_1_	458.3	194.1	4.8	24
Vitamin K_1_	451.4	199.2	4.8	32
Vitamin K_1_*	451.4	187.1	4.8	24
d7-MK-4	452.4	194.1	3.1	20
MK-4	445.3	227.1	3.1	24
MK-4*	445.3	187	3.1	20
d7-MK-7	656.5	194.1	7.9	32
MK-7	649.5	227.2	7.9	32
MK-7*	649.5	187	7.9	32

#### Stock solutions and quality controls

Stock solutions of the analytes (PK, MK-4, MK-7) and deuterated IS (d7-K_1_, d7-MK-4, d7-MK-7) were prepared by dissolving of each vitamin to the final concentration of 200 ng/mL and to the final concentration of 500 ng/mL for each deuterated IS in ethanol. Calibration standards were prepared from stock solutions and pooled serum from healthy volunteers. The pooled serum was exposed to UV light for at least 24 h due to photodegradation of endogenousvitamin K. After 24 h, the pooled serum was measured to demonstrate the elimination of endogenous vitamin K. The calibration range was from 0.03 to 10 ng/mL: 0.03; 0.05; 0.1; 0.3; 0.6; 1.0; 1.5; 2.0; 4.0; 10.0 ng/mL, respectively. Stock and working standards of analytes and deuterated IS were stored at −20 °C. The pooled serum was spiked with the stock solution of the analytes to obtain low- and high-quality control (QC) samples in concentrations of 0.5 and 1.5 ng/mL. Prepared QC samples were stored at −80 °C.

#### Subject and study design

Volunteers were determined to be healthy according to their medical history, physical examination and standard laboratory procedures. Samples were measured from 191 volunteers (51.2 ± 16.2 years (mean ± SD)), with 149 participants being female and 42 male. Ethical approval for this study was obtained from Ethics committee of University Hospital Motol (Ref.No.: EK-1422/13). Written informed consent was obtained from the participants at recruitment prior to their inclusion in the study.

#### Sample preparation

The samples were collected in five mL Greiner Tubes (Vacuette) containing a clot activator. After centrifugation (4 °C, 3,220×*g*, 10 min), the serum was transferred to plastic tubes, protected from light and stored at −80 °C until analysis. Before analysis, 500 μL aliquots of serum were transferred into glass tubes and spiked with 10 μL of each ISs. Two mL of ethanol were added due to the denature of proteins. After briefly mixing, four mL of n-hexane were added. After mixing for 5 min, the solution was centrifuged at 2,147×*g* for 10 min. The supernatant was transferred to a new covered glass tube, and the bottom layer was re-extracted by another four mL of n-hexane. Mixing and centrifugation conditions were the same as the first extraction. Both supernatants were collected and evaporated under a stream of nitrogen at 50 °C. The lipid extract was dissolved by two mL of n-hexane, and then a solid phase extraction (SPE) was required. The sample was applied to the silica Sep-Pak extraction cartridge (500 mg/3mL, Waters, USA) connected to a Visiprep SPE Vacuum Manifold (Sigma-Aldrich, Munich, Germany), which was washed prior with three mL diethyl ether:n-hexane (1:1) and then 3 × 3 mL of n-hexane. After the sample application, the cartridge was washed with two mL of n-hexane and then was washed again with 3 × 3 mL of n-hexane. The sample was eluted with 3 × 3 mL diethyl ether:n-hexane (3:97). The eluate was evaporated under nitrogen. The residue was reconstituted in 65 μL isopropanol. The prepared sample was transferred to amber micro-vials, capped and placed into the cooled autosampler rack. The aliquot of four μL was automatically injected into the system. The samples had to be protected from light during their preparation.

## Results

Our LC–MS/MS method for analysis of K_1_, MK-4 and MK-7 in human serum has been successfully validated following the criteria of the U.S. Food and Drug Administration (FDA) (2013). Calibration curves were established by plotting the peak area ratios K_1_, MK-4 and MK-7 against IS at 10-point concentrations. Pooled serum samples exposed to UV light were spiked with 0.03, 0.05, 0.1, 0.3, 0.6, 1.0, 1.5, 2.0, 4.0 and 10.0 ng/mL to obtain calibration curves. The assays were linear across the whole range of concentrations with mean correlation coefficients *R*^2^ of 0.980 for K_1_, 0.994 for MK-4 and 0.978 for MK-7. The first point of calibration curves (0.03 ng/mL) corresponds to the lower limit of quantification (LLOQ). Limits of detection (LoD) were determined by a further dilution of the first calibration point (LLOQ). Concentrations of 0.0019 ng/mL for K_1_ and MK-7 and 0.00375 ng/mL for MK-4 were obtained.

The intra- and inter-assay variation was determined using three concentration levels of QC samples: 0.05, 0.5 and 2.5 ng/mL. Intra-assay precision was obtained from 10 replicates measured in a single series and inter-assay imprecision from 25 different assays over a period of 6 weeks. The intra-assay coefficients of variation (CVs) were 10.4%, 3.2% and 2.3% for K_1_; were 14.3%, 3.2% and 6.7% for MK-4; and were 11.1%, 6.0% and 7.0% for MK-7. The inter-assay CVs were 12.8%, 11.3% and 7.4% for K1; were 15.2%, 9.2% and 8.7% for MK-4; and were 13.2%, 11.1% and 7.2% for MK-7.

**Figure 1:**
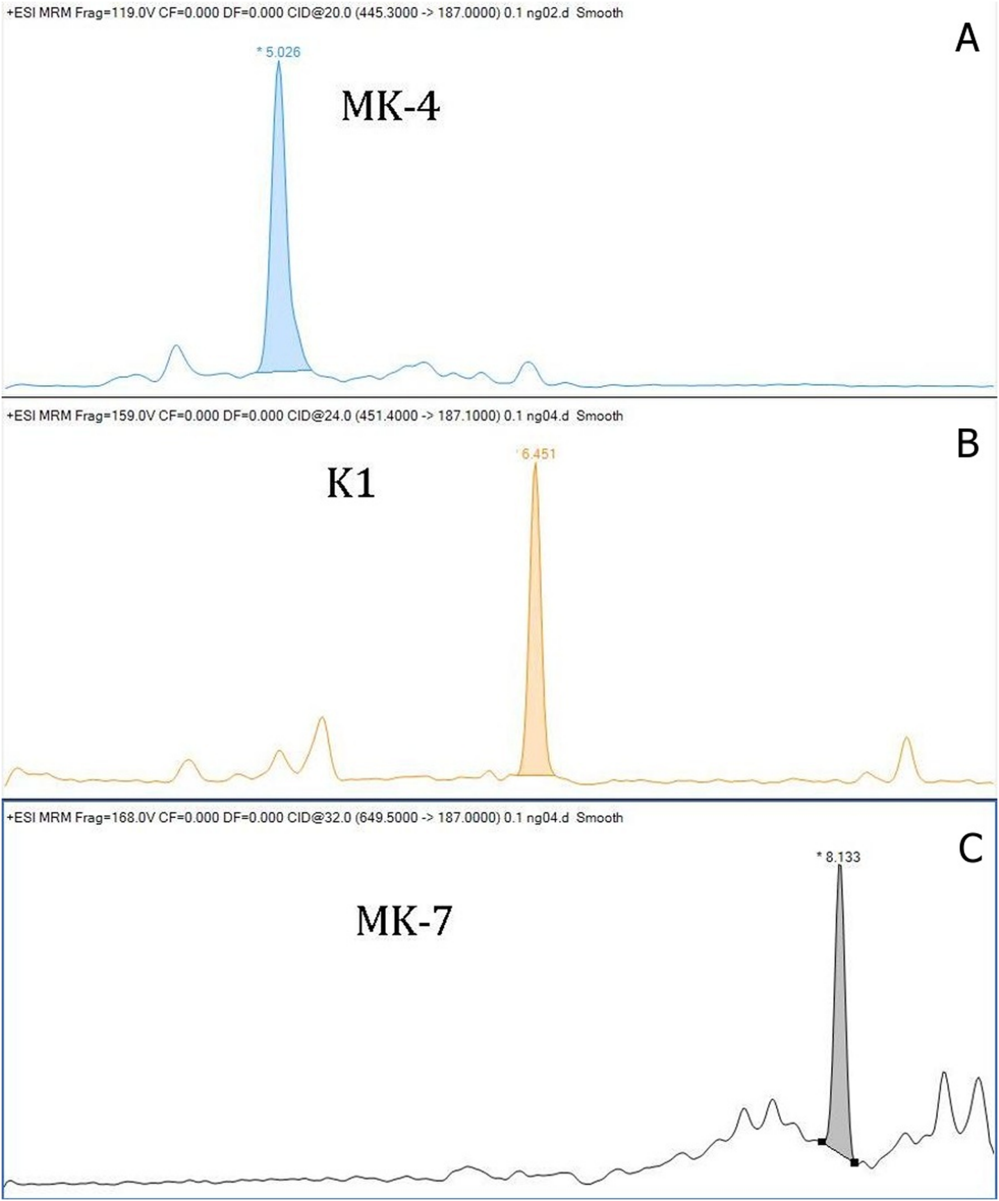
Chromatograms of vitamin K (A—chromatogram of MK-4, B—chromatogram of K1, C—chromatogram of MK-7).

The within-day accuracy was expressed by calculating the bias between observed and theoretical concentrations, and for K_1_ were 10.2%, 3.5% and 4.9%; for MK-4 were 10.7%, 0.5% and 6.0%; and for MK-7 were 6.7%, 2.0% and 6.6%. The recoveries for K_1_ were 102.6–108.3%, for MK-4 were 94.0–108.7, and for MK-7 were 100.6–106.7%. Chromatograms of K1, MK-4 and MK-7 obtained by LC–MS/MS are shown in Fig. 1. The stability of vitamins was tested. We tested the stability of vitamins in the fridge (2–8 °C) for 7 days, and the stability in the freezer for 3 months. Our results showed that the vitamins were not stable in the fridge. We noted mean decreases of 51.6% for MK-4, 16.8% for K_1_, and 23.0% for MK-7 after 7 days storage of serum in the refrigerator. The vitamins stored in the freezer were stable throughout the storage. Mean decreases of all vitamins were lower than 1%. The matrix effect was evaluated from vitamin free serum and water. The pretreatment procedure was performed, and after the SPE extraction the standards of each vitamin were added. The matrix factor was calculated in a range from −2.8% to +13% for all analytes. We performed a stability test of prepared sample stored in cooled autosampler for 168 h. There was a small decrease in vitamin K levels. The decreases did not exceed 12% against the original value in each form of vitamin K. We also tested an atmospheric pressure chemical ionization (ACPI) mode, but this type of ionization reported a lower sensitivity than the method with ESI. Our method with ESI is more convenient due to a routine laboratory mode.

We measured the vitamin K levels in a healthy Central European population. The group of patients consisted of 191 volunteers, 22% of them male, ages 51 ± 16 years (mean ± SD). Serum levels of K_1_, MK-4 and MK-7 from the healthy Central European population were: 0.03–2.19 (0.20) ng/mL, 0.05–2.79 (0.19) ng/mL, and 0.05–1.39 (0.09) ng/mL, respectively (min—max (median)).

No reference range for vitamin K_2_ has been published in the literature. What can be found in the literature are different reference ranges for vitamin K_1_. For example, Sadowski et al. (1989) published 0.11–1.01 ng/mL, and Kraemer stated 0.1–3.2 ng/mL in his publication. All our reference ranges were computed by an approved method (Harrell & Davis, 1982; Aytekin & Emerk, 2008; Horowitz, 2008; Tate, Yen & Jones, 2015), using the R statistical suite (R Core Team, 2015) with the package Hmisc (R Core Team, 2015; Harrell & Dupont, 2017). The gender difference was found significant only in the case of K_1_:0.044–1.357 ng/mL for women and 0.030–1.214 ng/mL for men. We performed Wilcoxon rank sum test with continuity correction, the *p*-value = 5.387e-06.The reference range calculated was 0.050–1.598 ng/mL for MK-4 for both genders, and 0.074–0.759 ng/mL for MK-7 for both women and men. The overview of our reference values is shown in Table 3.

**Table 3:** Reference ranges of three vitamin K's forms.

Phylloquinone	Menaquinone-4 *n* = 191	Menaquinone-7 *n* = 191
0.044–1.357 ng/mL (women, *n* = 149) 0.03–1.214 ng/mL (men, *n* = 42)	0.050–1.598 ng/mL	0.074–0.759 ng/mL

## Discussion

We describe a LC–MS/MS technique for determination of vitamin K_1_, MK-4 and MK-7 with low limits of quantification, which is suitable for the routine measurement of these vitamers in serum. This method enables us to find out the status of vitamin K. Vitamin K belongs to a group of liposoluble vitamins and is essential for bone metabolism and vascular health. Its key role was described in blood coagulation. Low vitamin K concentrations (especially K_2_) are associated with increased risks of osteoporotic fractures in the elderly, and vascular calcification (Schurgers et al., 2005; Fusaro et al., 2011). Vascular calcification is a pathological change found in atherosclerosis. In mice, it was established that a gene knockout of matrix Gla protein (MGP) caused death due to aortic calcification. In humans uncarboxylated MGP, which is an inactive form of MGP, is associated with the risk of cardiovascular disease and mortality. Osteoporosis is a pathological change of bone structure. Published studies have established a possible correlation between the level of uncarboxylated osteocalcin (ucOC) and the risk of fractures. Some recent studies have shown that vitamin K_2_ could be used as a novel adjuvant therapy to improve glycemic control and the quality of life in diabetics. Potential anticancer effects of the vitamin K family are the newest interest point for many researchers. Vitamin K_2_ may safely inhibit the growth and invasion of human hepatocellular carcinoma and pancreatic carcinoma. Another vitamin K dependent protein, Gas6, plays an important role in the central nervous system. Vitamin K may have some role in the pathogenesis of Alzheimer’s disease. Vitamin K_1_ intake in the elderly with Alzheimer’s disease was shown to be significantly lower than in the control group. Vitamin K_1_ deficiency may cause bleeding problems in newborns (Torri et al., 2016; Schwalfenberg, 2017).

Vitamin K status can be assessed by indirect functional assays, such as prothrombin time, international normalized ratio (INR) or by measuring undercarboxylated proteins (for example osteocalcin or MGP). Patients treated with warfarin are routinely monitored for INR in order to minimize the risk of bleeding. This test provides no information about the levels of vitamin K_2_, but patients treated with warfarin are at risk for increased susceptibility to vascular calcification and fractures caused by a reduction in the levels of vitamin K dependent carboxylated enzymes, MGP, and bone Gla-protein or osteocalcin. These patiens are in a greater risk of developing osteoporosis and fractures, and they could be supplemented by vitamin K_2_. It would be beneficial to monitor the levels of vitamin K (Fusaro et al., 2011).

It has been published that it is much easier to find out the status of vitamin K by measuring under-carboxylated vitamin K dependent proteins, where the analytical methods are less complicated. However, ucOC is also affected by vitamin D, therefore the levels of ucOC may not reflect the actual concentration of vitamin K properly. We also performed another research showing that proteins induced in the absence of vitamin K (PIVKA II) did not reflect the levels of MK-7 in patients treated with 45 µg/day of MK-7 for at least for 3 months. PIVKA could be a sensitive marker of mild vitamin K deficiency, but with a slow response. The next indirect assays were measurements of urinary vitamin K metabolites such as 7C-aglycone and 5C-aglycone which belong to non-invasive assays. Results indicated a good relationship between urinary metabolites with dietary vitamin K intake. Urinary vitamin K metabolites were mainly tested in pediatric populations. The main disadvantages of the indirect assays of vitamin K status were slow response to vitamin K deficiency and non-differentiation of vitamin K forms (Fusaro et al., 2017).

In recent years, research on LC–MS/MS methods have found them to be more sensitive and specific than HPLC methods (Riphagen et al., 2016; Tsugawa et al., 2006; Karl et al., 2014; Song et al., 2008). Quantifying levels of vitamin K is quite difficult due to the low concentration of these vitamins in body fluids. However, HPLC methods have been published more than LC–MS/MS techniques (Klapkova et al., 2018). The most common HPLC method for determination of vitamin K is fluorescent or electrochemical detection (Klapkova et al., 2018).

We developed and described the LC–MS/MS method for quantitation of K_1_, MK-4 and MK-7. The method has particular demands for the preparation of samples, but this is necessary, especially for the detection of low levels of MK (especially the level of MK-7). Riphagen et al. (2016) published a LC–MS method with ACPI for determination of PK and two forms of MK using a simplified pre-treatment sample procedure. The limit of quantification of MK-7 was 2.2 ng/mL. Intra- and inter-assays of MK-7 were published only for the high QC level (5.18 ng/mL). Therefore, their method is definitely not suitable for monitoring the levels in a healthy population, where the levels of MK-7 are much lower. These authors did not use SPE in preparation of the sample and they achieved a very poor limit of quantification of MK-7. Based on this study especially, it is evident that SPE is necessary for the removal of interfering substances, which mainly affect MK-7. Our LoD for each form of vitamin K is low, and it is very important for clinical use.

Presently, there is only one commercially available kit used only for the direct determination of K_1_, and it is from Immundiagnostik (Bensheim, Germany) with an LoD 0.15 ng/mL. The kit is for HPLC system using post-column zinc reduction and fluorescence detection. We performed some experiments using this kit, but we were not able to separate peaks of K_1_ and IS sufficiently.

Tsugawa et al. (2006) determined levels of K_1_, MK-4, and MK-7 using liquid chromatography with ACPI and tandem mass spectrometry (LC–APCI–MS). Their subjects were divided into three groups by age; the first group was premenopausal women 30–49 years old, the second group was postmenopausal women 50–69 years old, and the third group was postmenopausal women older than 70 years old. All women came from the region of Nagano, Japan, where the traditional meal “natto” is rich in MK-7. All groups had higher levels of MK-7 compared to our subjects: they had 4.96 ± 6.96 ng/mL in the first group, 8.42 ± 11.44 ng/mL in the second group and 4.21 ± 6.81 ng/mL in the third group. However, our subjects achieved a higher level of MK-4 compared to the subjects of Tsugawa et al. (2006). The same principle method was used by Karl et al. (2014) and Song et al. (2008). Karl et al. (2014) determined K_1_ and up to 10 forms of vitamin K_2_ (MK-4 to MK-13) in faeces, serum and food. The serum levels of K_1_ were 0.45 ± 0.27 ng/mL in the group consuming refined grain and 0.54 ± 0.32 ng/mL in the group consuming whole grain. Serum levels of MK-4 to MK-13 were under LoD. Song et al. (2008) determined K_1_ with LoD 0.3 ng/mL. This LoD is quite high considering the fact they used the LC–ACPI–MS method. An LC–MS/MS method was published for determination of K_1_, MK-4 and MK-7 using isotopically labeled vitamins as IS. Klapkova et al. (2018) published a HPLC method using fluorescence detection and post-column zinc reduction with LoD 0.03 ng/mL for K_1_ and MK-7; and 0.04 ng/mL for MK-4. The serum levels of PK, MK-4 and MK-7 in postmenopausal women with osteoporosis and without osteoporosis were published. Serum levels of K_1_, MK-4 and MK-7 in women with osteoporosis were: 0.43 ± 0.39, 0.89 ± 0.29 and 1.00 ± 1.02 ng/mL, respectively. Serum levels of K_1_, MK-4 and MK-7 in women without osteoporosis were: 0.49 ± 0.40, 0.83 ± 0.27 and 1.19 ± 1.08 ng/mL, respectively.

Suttie (1992) published levels of vitamins K in normal subjects in the UK. There were 11 young normal subjects with K_1_ and MK-7 levels in concentrations of 0.51 ± 0.37 and 0.29 ± 0.18 ng/mL, respectively (mean ± SD). In elderly normal subjects (*n* = 17), the levels of PK and MK-7 were 0.60 ± 0.28 and 0.33 ± 0.17 ng/mL, respectively (mean ± SD). Both of the MK-7 subject levels are very similar to our results 0.25 ± 0.21 ng/mL (mean ± SD), but there are high levels of vitamin K_1_. Yan et al. (2004) published levels of K_1_ in older men (*n* = 86) and women (*n* = 92) in China. In this paper, they did not determine the levels of menaquinones. The levels of PK were 0.85 ± 0.99 and 1.12 ± 1.45 ng/mL, respectively, in men and women (mean ± SD). They also had higher levels of K_1_ than our results. Booth et al. (2004) published a large study of the male population (*n* = 741) for the determination of K_1_. The level of K_1_ was 0.69 ± 0.90 ng/mL. Kamao et al. (2005) and Suhara et al. (2005) published studies with healthy subjects in Japan. Kamao et al. (2005) determined K_1_, MK-4 and MK-7 in 20 healthy subjects. Levels of vitamins K were 1.81 ± 1.11, 0.15 ± 0.17 and 16.27 ± 20.58 ng/mL for K_1_, MK-4 and MK-7 (mean ± SD), respectively. In this case, there were levels of MK-4 lower than our results, while levels of MK-7 were several times higher than our results. This could be present in the traditional Japanese meal *nattō*, which is rich in MK-7 as shown in the Tsugawa article (2006). Suhara et al. (2005) published similar results of healthy population as Kamao et al. (2005). Levels of vitamin K in healthy subjects (*n* = 20) were 1.22 ± 0.57, 0.39 ± 0.46 and 6.37 ± 7.45 ng/mL for K_1_, MK-4 and MK-7 (mean ± SD), respectively. The serum levels of vitamin K reported in previous studies are shown in Table 4.

**Table 4:** Published levels of vitamin K in serum.

References	Region		Phylloquinone (ng/mL)	Menaquinone-4 (ng/mL)	Menaquinone-7 (ng/mL)
Tsugawa et al. (2006)	Japan	Premenopausal women 30–49 years (*n* = 52) Postmenopausal women 50–69 years (*n* = 208) Postmenopausal women ≥70 years (*n* = 136)	1.52 ± 1.02 1.74 ± 1.29 1.29 ± 1.09	0.07 ± 0.14 0.10 ± 0.19 0.09 ± 0.15	4.96 ± 6.93 8.42 ± 11.44 4.21 ± 6.81
Riphagen et al. (2016)	Netherlands	Patients after kidney transplant (*n* = 60)	0.61 ± 0.21	0.09 ± 0.01	<LoD (2.86)
Klapkova et al. (2018)	Czech Republic	Postmenopausal women with osteoporosis (*n* = 192) Postmenopausal women without osteoporosis (*n* = 158)	0.43 ± 0.39 0.49 ± 0.40	0.89 ± 0.29 0.83 ± 0.27	1.00 ± 1.02 1.19 ± 1.08
Suttie (1992)	UK	Young population (*n* = 11) Older population (*n* = 17)	0.51 ± 0.37 0.60 ± 0.28	not measured	0.29 ± 0.18 0.33 ± 0.17
Yan et al. (2004)	China	Older men (*n* = 86) Older women (*n* = 92)	0.85 ± 0.99 1.12 ± 1.45	not measured	not measured
Booth et al. (2004)	USA	Older men (*n* = 741)	0.69 ± 0.90	not measured	not measured
Kamao et al. (2005)	Japan	Healthy population (*n* = 20) Patients with osteoporosis (*n* = 10)	1.81 ± 1.11 0.62 ± 0.25	0.15 ± 0.17 46.83 ± 46.41	16.27 ± 20.58 4.18 ± 6.28
Suhara et al. (2005)	Japan	Healthy population (*n* = 20)	1.22 ± 0.57	0.39 ± 0.46	6.37 ± 7.45

At this time, HPLC and LC–MS/MS methods are used for determination of vitamin K levels. Both types of methods can be used with sufficient pre-purification of the samples. The sufficient pre-purification of the sample is necessary for achievement of low LoD. No immunoassay for determination of vitamin K has been published.

## Conclusions

Our LC–MS method presented here is applicable for three forms of vitamin K (K_1_, MK-4 and MK-7) determination. Due to the performance of solid-phase extraction, the LoD are low, and the method is sensitive and robust enough to quantify phylloquinone and two types of menaquinones in human serum. In order to improve the outcomes of some medical treatments, especially those related to bone metabolism and inhibition of vascular calcification, it is recommended to measure the levels of phylloquinone and menaquinones in serum or plasma.

Direct measurement of serum phylloquinone and two types of menaquinones is considered to be more appropriate marker than indirect markers determining vitamin K deficiency (e.g., uncarboxylated MGP or ucOC). Determination of vitamin K levels is more suitable due to the early response of the organism. So far, the biggest problem is the lack of data of RDIs and reference ranges for all forms of vitamin K, which makes assessing the status of vitamin K more difficult. The difficult pre-purification sample process is time-consuming and requires high-tech devices. Therefore the measurement of this vitamin is only possible in a specialized laboratory. The low toxicity and beneficial effects of MK-7 on both bone metabolism and vascular calcification should be strongly considered as a therapeutic agent in the treatment of several diseases. Vitamin K could additionally play a role in the treatment of cancer or in the prevention of diabetes mellitus. Determination of vitamin K levels would be beneficial for patients undergoing warfarin therapy.

## Supplemental Information

10.7717/peerj.7695/supp-1 Supplemental Information 1 Raw data.

10.7717/peerj.7695/supp-2 Supplemental Information 2 Chromatogram of analytical standards.

## Additional Information and Declarations

### Data Availability

The following information was supplied regarding data availability: The raw measurements are available in Dataset S1. The raw data shows all measured values. The chromatograms of the analytical standards for each vitamer of vitamin K are also available in the Supplemental Files.
