# Supplementary material for: LC–MS/MS quantitative analysis of phylloquinone, menaquinone-4 and menaquinone-7 in the human serum of a healthy population
Source: PeerJ. 2019 Sep 19;7:e7695. doi: 10.7717/peerj.7695 (PMC6754977; doi:10.7717/peerj.7695)
Supplement: Supplemental Information 1 [file peerj-07-7695-s001.doc]

| No. | Sex | MK-4 ng/ml | K1 ng/ml | MK-7 ng/ml |
| --- | --- | --- | --- | --- |
|
| 1 | F | 0,11 | 0,21 | 0,16 |
| 2 | F | 0,05 | 0,32 | 0,10 |
| 3 | F | 0,05 | 1,46 | 0,33 |
| 4 | F | 1,98 | 0,39 | 0,19 |
| 5 | F | 0,24 | 0,61 | 0,10 |
| 6 | F | 0,23 | 0,66 | 0,28 |
| 7 | F | 1,47 | 0,26 | 0,19 |
| 8 | F | 1,76 | 0,19 | 0,24 |
| 9 | F | 0,22 | 0,28 | 0,33 |
| 10 | F | 1,85 | 0,52 | 0,22 |
| 11 | F | 0,09 | 0,47 | 0,29 |
| 12 | F | 0,08 | 1,12 | 0,34 |
| 13 | F | 0,12 | 2,05 | 0,50 |
| 14 | F | 0,09 | 0,06 | 0,18 |
| 15 | F | 0,05 | 0,76 | 0,30 |
| 16 | F | 0,10 | 0,25 | 0,31 |
| 17 | F | 0,10 | 0,19 | 0,13 |
| 18 | F | 0,08 | 0,21 | 0,22 |
| 19 | F | 1,40 | 0,18 | 0,31 |
| 20 | F | 0,74 | 0,29 | 0,15 |
| 21 | F | 0,10 | 0,34 | 0,20 |
| 22 | F | 1,66 | 0,44 | 0,22 |
| 23 | F | 0,07 | 0,24 | 0,14 |
| 24 | F | 0,05 | 0,29 | 0,15 |
| 25 | F | 0,05 | 0,35 | 0,06 |
| 26 | F | 0,05 | 0,60 | 0,23 |
| 27 | F | 0,05 | 0,31 | 0,23 |
| 28 | F | 0,21 | 0,25 | 0,57 |
| 29 | F | 0,05 | 0,17 | 0,29 |
| 30 | F | 0,05 | 0,64 | 0,29 |
| 31 | F | 0,05 | 0,13 | 0,26 |
| 32 | F | 0,05 | 0,11 | 0,19 |
| 33 | F | 0,39 | 0,05 | 0,16 |
| 34 | F | 1,15 | 0,55 | 0,20 |
| 35 | F | 0,05 | 0,10 | 0,10 |
| 36 | F | 0,62 | 0,45 | 0,13 |
| 37 | F | 1,11 | 2,19 | 0,31 |
| 38 | F | 1,07 | 0,20 | 0,41 |
| 39 | M | 0,95 | 0,70 | 0,20 |
| 40 | F | 0,45 | 0,90 | 0,27 |
| 41 | F | 0,05 | 0,43 | 0,20 |
| 42 | F | 0,44 | 0,30 | 0,21 |
| 43 | F | 0,05 | 0,24 | 0,27 |
| 44 | F | 0,17 | 0,83 | 0,16 |
| 45 | F | 0,05 | 0,72 | 0,26 |
| 46 | F | 0,15 | 0,42 | 0,12 |
| 47 | F | 0,05 | 0,33 | 0,12 |
| 48 | F | 0,27 | 0,16 | 0,21 |
| 49 | F | 0,83 | 0,17 | 0,31 |
| 50 | F | 0,05 | 0,20 | 0,18 |
| 51 | F | 0,05 | 0,13 | 0,27 |
| 52 | F | 0,14 | 0,42 | 0,23 |
| 53 | F | 0,44 | 0,83 | 0,17 |
| 54 | F | 0,05 | 0,17 | 0,12 |
| 55 | F | 0,09 | 0,35 | 0,34 |
| 56 | M | 0,20 | 0,14 | 0,20 |
| 57 | F | 0,05 | 0,03 | 0,07 |
| 58 | F | 0,57 | 0,24 | 0,18 |
| 59 | F | 0,37 | 0,24 | 0,14 |
| 60 | F | 0,05 | 0,09 | 0,05 |
| 61 | F | 0,34 | 0,16 | 0,18 |
| 62 | F | 0,05 | 0,05 | 0,11 |
| 63 | M | 0,21 | 0,21 | 0,24 |
| 64 | F | 0,69 | 0,04 | 0,10 |
| 65 | M | 0,05 | 0,08 | 0,16 |
| 66 | M | 0,07 | 0,08 | 0,10 |
| 67 | M | 0,30 | 0,07 | 0,19 |
| 68 | M | 0,44 | 0,06 | 0,22 |
| 69 | F | 0,11 | 0,09 | 0,06 |
| 70 | F | 0,95 | 0,11 | 0,07 |
| 71 | F | 0,11 | 0,40 | 0,06 |
| 72 | F | 0,98 | 0,08 | 0,05 |
| 73 | M | 0,77 | 0,09 | 0,35 |
| 74 | M | 0,05 | 0,13 | 0,08 |
| 75 | M | 0,06 | 0,08 | 0,32 |
| 76 | M | 0,05 | 0,10 | 0,13 |
| 77 | F | 0,05 | 0,10 | 0,08 |
| 78 | F | 0,05 | 0,16 | 0,38 |
| 79 | M | 0,05 | 0,03 | 0,30 |
| 80 | M | 0,85 | 0,06 | 0,09 |
| 81 | M | 0,09 | 0,04 | 0,16 |
| 82 | F | 0,05 | 0,09 | 0,10 |
| 83 | F | 0,05 | 0,03 | 0,13 |
| 84 | F | 0,67 | 0,14 | 0,26 |
| 85 | M | 0,07 | 0,08 | 0,22 |
| 86 | F | 0,05 | 0,12 | 0,06 |
| 87 | F | 0,21 | 0,53 | 0,42 |
| 88 | F | 0,52 | 0,23 | 0,17 |
| 89 | M | 0,05 | 0,12 | 0,09 |
| 90 | M | 0,10 | 0,05 | 0,09 |
| 91 | M | 0,05 | 0,12 | 0,20 |
| 92 | M | 0,60 | 0,05 | 0,14 |
| 93 | F | 0,75 | 0,14 | 0,42 |
| 94 | M | 0,05 | 0,44 | 0,06 |
| 95 | M | 0,14 | 0,15 | 0,17 |
| 96 | M | 0,85 | 0,04 | 0,07 |
| 97 | F | 0,24 | 0,15 | 0,11 |
| 98 | M | 0,21 | 0,24 | 0,36 |
| 99 | F | 0,46 | 0,18 | 0,17 |
| 100 | M | 1,06 | 0,03 | 0,20 |
| 101 | F | 0,05 | 0,31 | 0,42 |
| 102 | M | 0,05 | 0,05 | 0,06 |
| 103 | F | 1,79 | 0,11 | 0,21 |
| 104 | F | 1,49 | 0,09 | 0,27 |
| 105 | M | 0,07 | 0,05 | 0,09 |
| 106 | F | 0,09 | 0,11 | 0,12 |
| 107 | F | 2,79 | 0,11 | 0,47 |
| 108 | M | 1,35 | 0,04 | 0,43 |
| 109 | F | 1,10 | 0,07 | 0,15 |
| 110 | F | 0,05 | 0,04 | 0,09 |
| 111 | F | 0,05 | 0,04 | 0,21 |
| 112 | M | 0,05 | 0,03 | 0,07 |
| 113 | M | 0,05 | 0,08 | 0,16 |
| 114 | F | 0,05 | 0,30 | 0,71 |
| 115 | F | 0,05 | 0,05 | 0,29 |
| 116 | F | 0,24 | 1,92 | 0,25 |
| 117 | F | 0,05 | 0,14 | 0,28 |
| 118 | F | 0,91 | 0,18 | 0,10 |
| 119 | F | 0,16 | 0,70 | 1,31 |
| 120 | F | 0,09 | 0,10 | 0,12 |
| 121 | F | 0,10 | 0,08 | 0,83 |
| 122 | F | 0,09 | 0,32 | 0,20 |
| 123 | F | 0,18 | 0,21 | 0,16 |
| 124 | M | 0,28 | 0,10 | 0,17 |
| 125 | F | 0,13 | 0,10 | 0,44 |
| 126 | F | 0,05 | 0,14 | 0,14 |
| 127 | F | 0,10 | 1,94 | 0,39 |
| 128 | F | 0,10 | 0,19 | 0,37 |
| 129 | M | 0,46 | 0,13 | 0,22 |
| 130 | F | 0,08 | 0,73 | 0,17 |
| 131 | M | 0,09 | 0,11 | 0,15 |
| 132 | F | 0,05 | 0,08 | 0,06 |
| 133 | F | 0,05 | 0,60 | 0,18 |
| 134 | F | 0,08 | 0,56 | 0,26 |
| 135 | F | 0,05 | 0,12 | 0,10 |
| 136 | F | 0,08 | 0,16 | 0,09 |
| 137 | F | 0,07 | 0,41 | 0,12 |
| 138 | F | 0,14 | 0,21 | 0,37 |
| 139 | F | 0,05 | 0,18 | 0,14 |
| 140 | F | 0,05 | 0,24 | 0,15 |
| 141 | F | 0,12 | 0,16 | 0,42 |
| 142 | F | 0,05 | 0,20 | 0,19 |
| 143 | F | 0,06 | 0,27 | 0,17 |
| 144 | F | 0,09 | 0,48 | 0,20 |
| 145 | F | 0,07 | 0,12 | 0,13 |
| 146 | F | 0,08 | 0,13 | 0,21 |
| 147 | F | 2,38 | 0,12 | 0,28 |
| 148 | F | 0,20 | 0,20 | 0,17 |
| 149 | F | 0,09 | 0,93 | 0,16 |
| 150 | F | 0,06 | 0,31 | 0,49 |
| 151 | M | 0,05 | 0,36 | 0,20 |
| 152 | M | 0,20 | 1,08 | 0,29 |
| 153 | M | 0,05 | 0,18 | 0,29 |
| 154 | M | 0,20 | 0,38 | 0,14 |
| 155 | M | 0,35 | 0,14 | 0,11 |
| 156 | M | 0,18 | 0,36 | 0,15 |
| 157 | F | 0,14 | 0,22 | 0,48 |
| 158 | F | 0,18 | 0,41 | 0,11 |
| 159 | F | 0,37 | 0,22 | 0,09 |
| 160 | M | 0,06 | 0,17 | 0,28 |
| 161 | M | 0,18 | 1,35 | 0,49 |
| 162 | F | 0,11 | 0,28 | 0,17 |
| 163 | F | 0,05 | 0,11 | 0,10 |
| 164 | F | 0,06 | 0,50 | 0,15 |
| 165 | F | 0,05 | 0,11 | 0,15 |
| 166 | M | 0,10 | 0,05 | 0,10 |
| 167 | F | 0,10 | 0,91 | 0,12 |
| 168 | F | 0,08 | 0,71 | 0,30 |
| 169 | F | 0,10 | 0,18 | 0,20 |
| 170 | F | 0,13 | 0,14 | 0,18 |
| 171 | F | 0,43 | 1,69 | 0,26 |
| 172 | F | 0,05 | 0,18 | 0,26 |
| 173 | F | 0,05 | 0,41 | 0,26 |
| 174 | F | 0,05 | 0,21 | 0,27 |
| 175 | F | 0,05 | 0,07 | 0,59 |
| 176 | F | 0,05 | 0,52 | 1,30 |
| 177 | M | 0,05 | 0,67 | 0,23 |
| 178 | F | 0,05 | 0,35 | 0,55 |
| 179 | F | 0,05 | 0,16 | 0,19 |
| 180 | F | 0,27 | 0,53 | 0,15 |
| 181 | F | 0,49 | 0,34 | 0,17 |
| 182 | F | 0,05 | 0,67 | 0,90 |
| 183 | F | 1,20 | 0,35 | 0,18 |
| 184 | F | 0,07 | 0,21 | 0,20 |
| 185 | F | 0,09 | 0,77 | 1,20 |
| 186 | F | 0,05 | 0,45 | 0,22 |
| 187 | F | 0,05 | 0,18 | 1,00 |
| 188 | F | 0,08 | 0,35 | 1,39 |
| 189 | F | 0,05 | 0,26 | 0,32 |
| 190 | F | 0,05 | 0,36 | 0,11 |
| 191 | F | 0,05 | 0,27 | 0,18 |
