# Supplementary figures and images for: LC–MS/MS quantitative analysis of phylloquinone, menaquinone-4 and menaquinone-7 in the human serum of a healthy population
Source: PeerJ. 2019 Sep 19;7:e7695. doi: 10.7717/peerj.7695 (PMC6754977; doi:10.7717/peerj.7695)

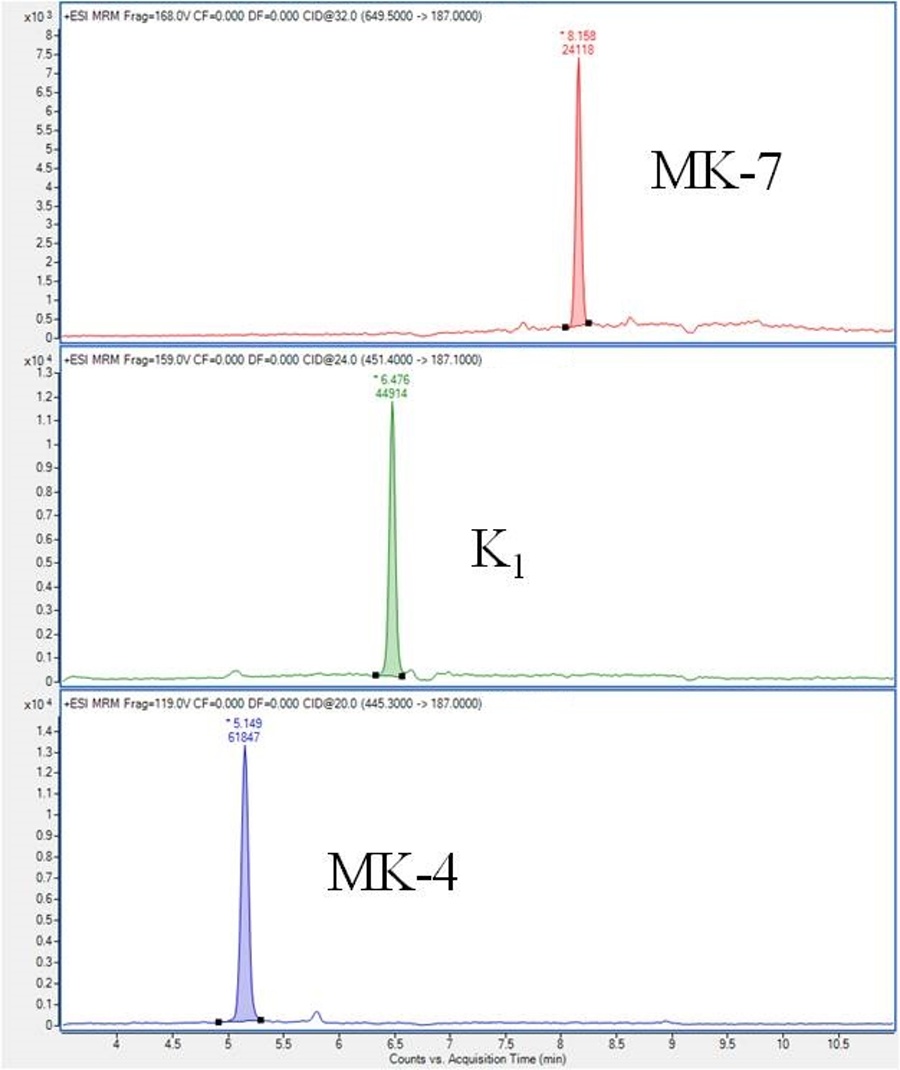

Supplement: Supplemental Information 2 [file peerj-07-7695-s002.jpg]
